# The recent research progress on traditional Chinese medicine application in Parkinson's disease: a review

**DOI:** 10.3389/fnagi.2026.1798695

**Published:** 2026-04-29

**Authors:** Qianying Feng, Yu Feng, Dan Li, Yaru Wang, Fengxia Chen, Binfeng Cheng, Junzheng Yang

**Affiliations:** 1 School of Life Sciences and Biotechnology, North Henan Medical University, Xinxiang, China; 2 Guangdong Nephrotic Drug Engineering Technology Research Center, Guangzhou, China

**Keywords:** Parkinson's disease, pathogenesis, risk factor, traditional Chinese medicine, underlying mechanism

## Abstract

**Background:**

Parkinson's disease (PD) is a type of neurodegenerative diseases with the characterized by static tremors, bradykinesia, and muscle rigidity. This is mainly caused by the degradation and death of dopaminergic neurons in the substantia nigra, severely affects the health of the elderly no matter in developing or developed countries. In recent years, Traditional Chinese medicine (TCM) has made significant advances in the treatment and application of PD with its unique advantages in recent years.

**Methods:**

A systematic search of the extant literature, published between 2021 and 2026, was conducted in the following databases: PubMed, Embase, Scopus, and Web of Science. The search was conducted using the keywords “Traditional Chinese Medicine”, “Parkinson's disease”, “preclinical application”, “clinical application”. We summarized the recent TCM application in PD including preclinical and clinical application and the underlying mechanisms were summarized, and discussed the possible application limitations.

**Results and conclusion:**

TCM exhibits significant potential for preclinical and clinical application in PD. The ongoing resolution of current application limitations, including the pharmacological substance basis and the mechanism of TCM, the establishment of quality standards for TCM, and the conducting the standardized clinical trials, will result in significant progress in the application of TCM in PD.

## Introduction

Parkinson's disease (PD) can be traced back to 1,817, when James Parkinson published the world's first detailed paper documenting PD (Tanner and Ostrem, 2024). PD is a type of neurodegenerative disease caused by the degeneration and death of dopaminergic neurons in the substantia nigra (Akyazi et al., 2024; Ben-Shlomo et al., 2024; Weintraub et al., 2022). Epidemiological survey data demonstrated that the global prevalence of PD continues is increasing. It is estimated that the global prevalence of PD will increase to 17 million people by 2040, and the number of PD patients in China will exceed 5 million. It has demonstrated that the prevalence of PD varies significantly according to geography, race, age, and gender (Gao et al., 2020). The statistical evidence has demonstrated that there was a close relationship between the process of aging and the pathogenesis of PD. The prevalence of PD among the Chinese population aged 65 and above in China is 1.7%, which is the significantly higher than in European and American countries (1%). The prevalence of PD is particularly high in elderly men than that in women, with a male-to-female approximately reached to 1.3–1.5:1. A range of factors, including the medical resources, diagnostic technology, and aging population, result in differences in the incidence rate and prevalence of PD between countries (Ahmad et al., 2023; Samii et al., 2004; Zhang et al., 2005). The incidence of PD is higher in developed countries than that in developing countries. Furthermore, the incidence rates in Europe and North America are significantly higher than those in Africa and Asia (Bhidayasiri et al., 2020; GBD 2021 Demographics Collaborators, 2021). Traditional Chinese medicine (TCM) has a long history, going back thousands of years, and has developed a unique theoretical foundation (Zhang and Fang, 2023). TCM emphasizes the unity and integrity of the human body, as well as its relationship with the natural world. When treating local diseases, it is necessary to consider the etiology and symptoms as a whole and adopt appropriate treatment methods (Liu et al., 2021). TCM boasts a wide variety of resources. To date, tens of thousands of TCMs have been discovered, which can be divided into several types, including Chinese herbal pieces, traditional Chinese patent medicines, prescriptions, herbal extracts, and formula granules. These have been successfully used to treat many types of diseases (Chen et al., 2024; Li et al., 2022; Wang and Yang, 2021; Yang et al., 2023). It has been proven that they have the effects of regulating oxidative stress, reducing inflammation, regulating immune function, affecting cell signaling, and gene expression (Duan et al., 2023; Li et al., 2023a; Yuan et al., 2026; Zhang et al., 2025). Many evidence has demonstrated that wide range of applications and excellent therapeutic efficacy of TCM in the treatment of PD (Chen J. et al., 2022; Muhammad et al., 2022). In this review, we summarized the recent TCM applications in PD including both preclinical and clinical application, as well as the underlying mechanisms, discussed the possible application limitations, and sought to provide a comprehensive understanding of the subject, as well as some useful clues for related researchers.

## Methodology

A comprehensive search of the extant literature, which has been published between 2021 and 2026, was conducted in the following databases: PubMed, Embase, Scopus by using the following keywords, including “Traditional Chinese Medicine”, “Parkinson's disease”, “preclinical application”, and “clinical application”.

## Results

### The pathogenesis and the risk factors of PD

**Figure 1:**
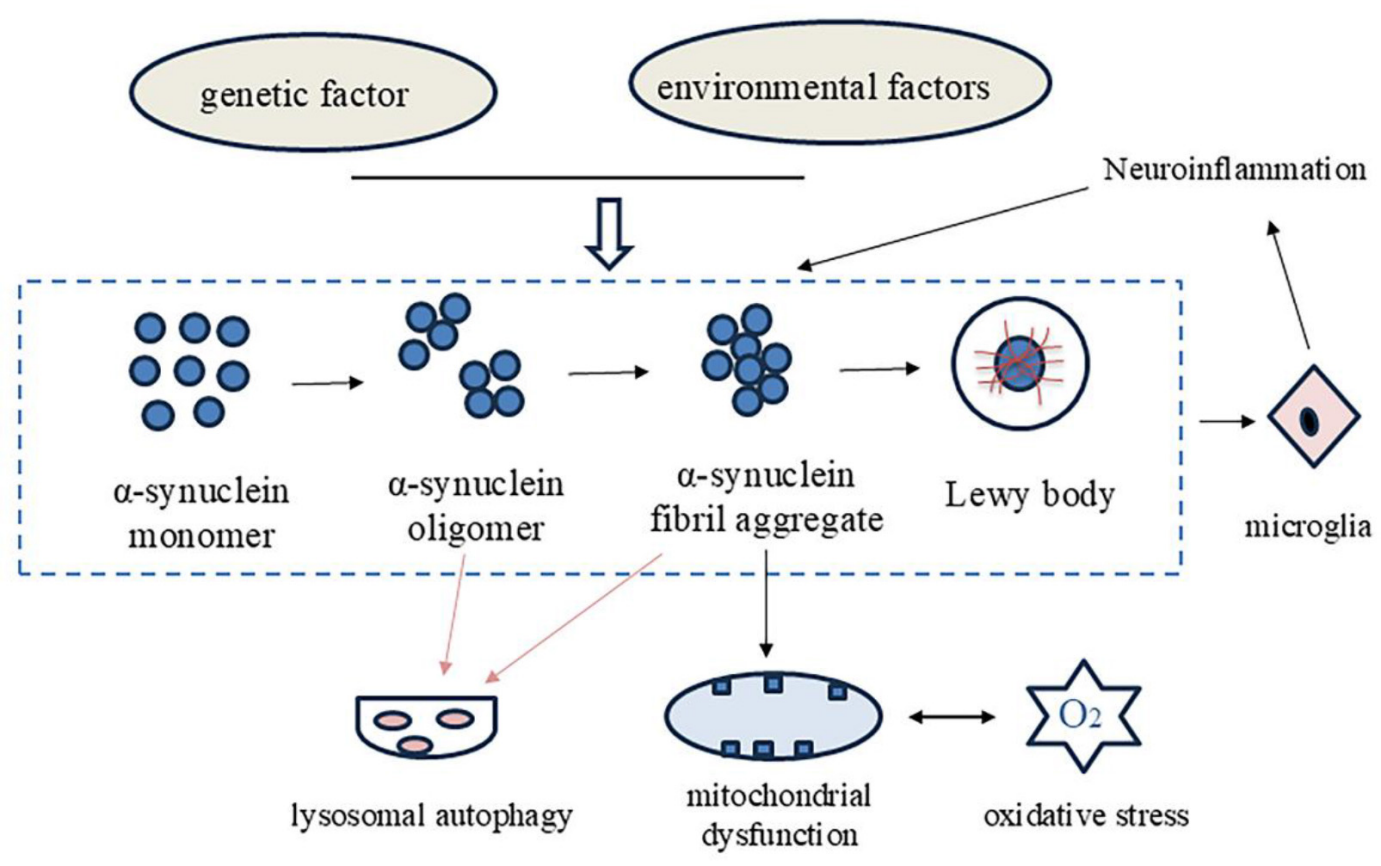
The summary of the pathogenesis of PD.

The pathogenesis of PD is a complex process, and research has identified a number of key factors that contribute to its development. These include the imbalance of alpha synuclein, mitochondrial dysfunction, oxidative stress, neuroinflammation, and the gut microbiota (gut brain axis; Chen J. et al., 2022; Dionísio et al., 2021; Malpartida et al., 2021; Zhang et al., 2023). Alpha synuclein is a presynaptic neuronal protein that exists in a balance between soluble and membrane-bound forms in neurons. However, in the brains of PD patients, an imbalance of alpha synuclein can lead to the formation of abnormal soluble oligomers. In addition to having an effect on the aggregation characteristics of the protein, an imbalance of alpha synuclein can also exacerbate mitochondrial dysfunction, oxidative stress, neuroinflammatory activation, and other reactions (Chen R. et al., 2022; Lv et al., 2023). The loss and mutation of mitochondrial DNA accumulate with age, leading to a continuous decline in mitochondrial function, Aging can lead to organelle damage and cellular dysfunction, further affecting PD which is closely related to the loss of neurons in the substantia nigra of PD patients, and aging can lead to organelle damage and cellular dysfunction, further affecting PD (Hinkle et al., 2022; Ma et al., 2025). The evidence demonstrated that there is a strong bidirectional interaction between the gut, its microbiota, and the brain, commonly known as the microbiota-gut-brain axis. This has important implications for brain health. Dietary modifications and fecal microbiota transplantation have been identified to improve the symptoms of PD patients by targeting gut microbiota (Zhao et al., 2021; Socała et al., 2021; Figure 1).

**Figure 2:**
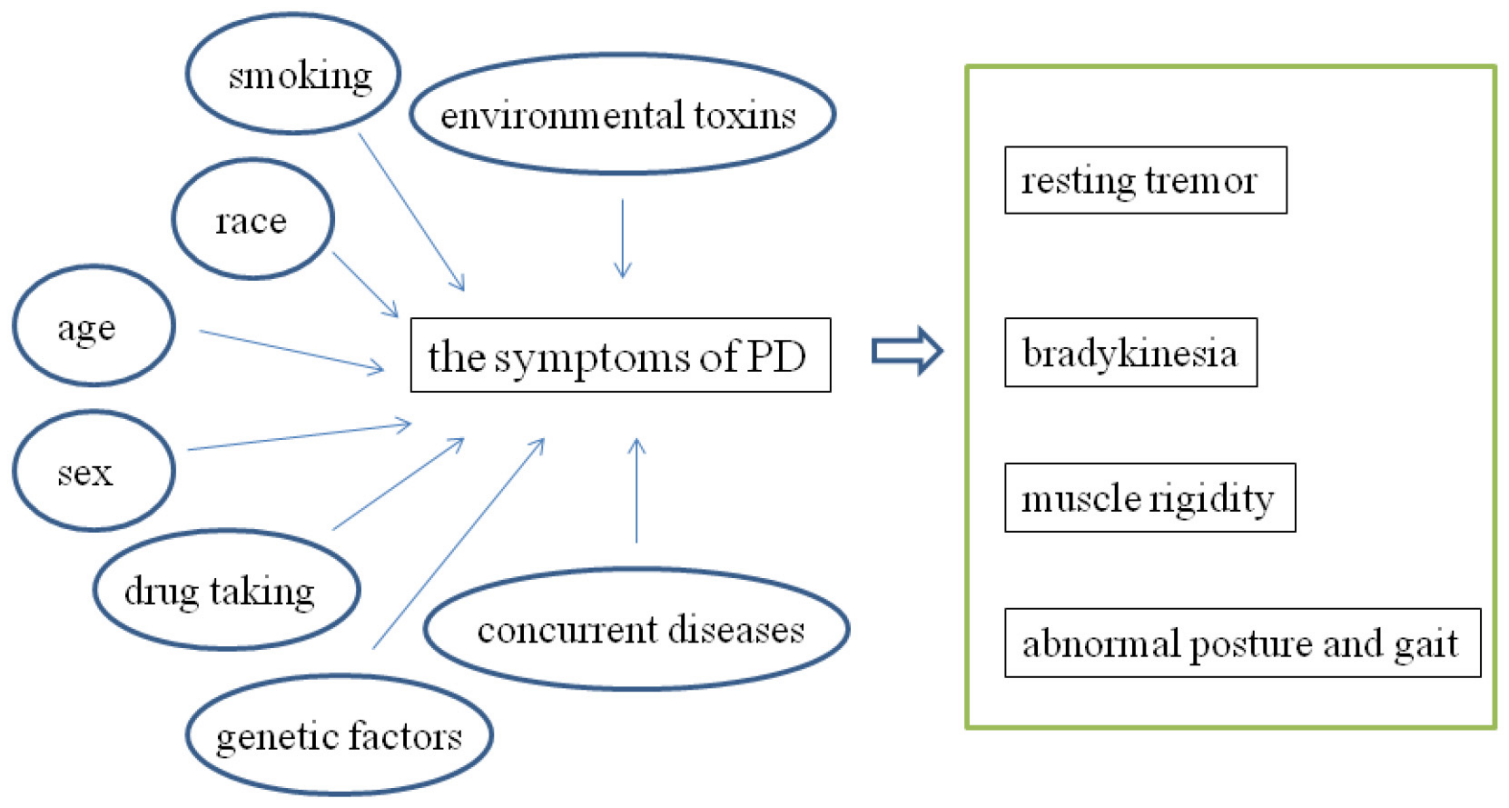
The summary of the primary risk factors of PD.

PD is a type of neurodegenerative diseases which is associated with the degeneration and death of dopaminergic neurons in the substantia nigra (Deng et al., 2025). The main symptoms of PD in clinic include resting tremor, muscle rigidity, bradykinesia, and abnormal posture and gait (Stamenović et al., 2024). There are several types of risk factors which promote the pathogenesis of PD have been identified, including race, age, sex, genetic factors, smoking, drug taking, environmental toxins, and several kinds of concurrent diseases (Ascherio and Schwarzschild, 2016; Grotewold and Albin, 2024; Figure 2). Statistical evidence demonstrated that the incidence rate is highest among white people, followed by yellow people, and the lowest among black people. Research has shown that the incidence of PD is closely related to age (Siddiqi et al., 2025). The survey data demonstrated that the incidence rate of PD is 0.01% at the age of 50, and it will reach to 0.2% at the age of 80. Numerous studies have demonstrated that there is a higher incidence of PD in men than that in women (Cattaneo and Pagonabarraga, 2025). The study demonstrated that MPTP exists in the illegal synthetic drug MPPP, which can specifically damage dopaminergic neurons in the substantia nigra. Amphetamine and methamphetamine, the main components of the drug, can also increase the incidence of PD. Fish vine ketone, paraquat, organic solvents, wood preservatives, and special heavy metals present in the living environment, depression patients, and diabetes patients can also increase the risk of PD (Atterling Brolin et al., 2025; Newell et al., 2025).

### The preclinical applications of TCM in PD

**Figure 3:**
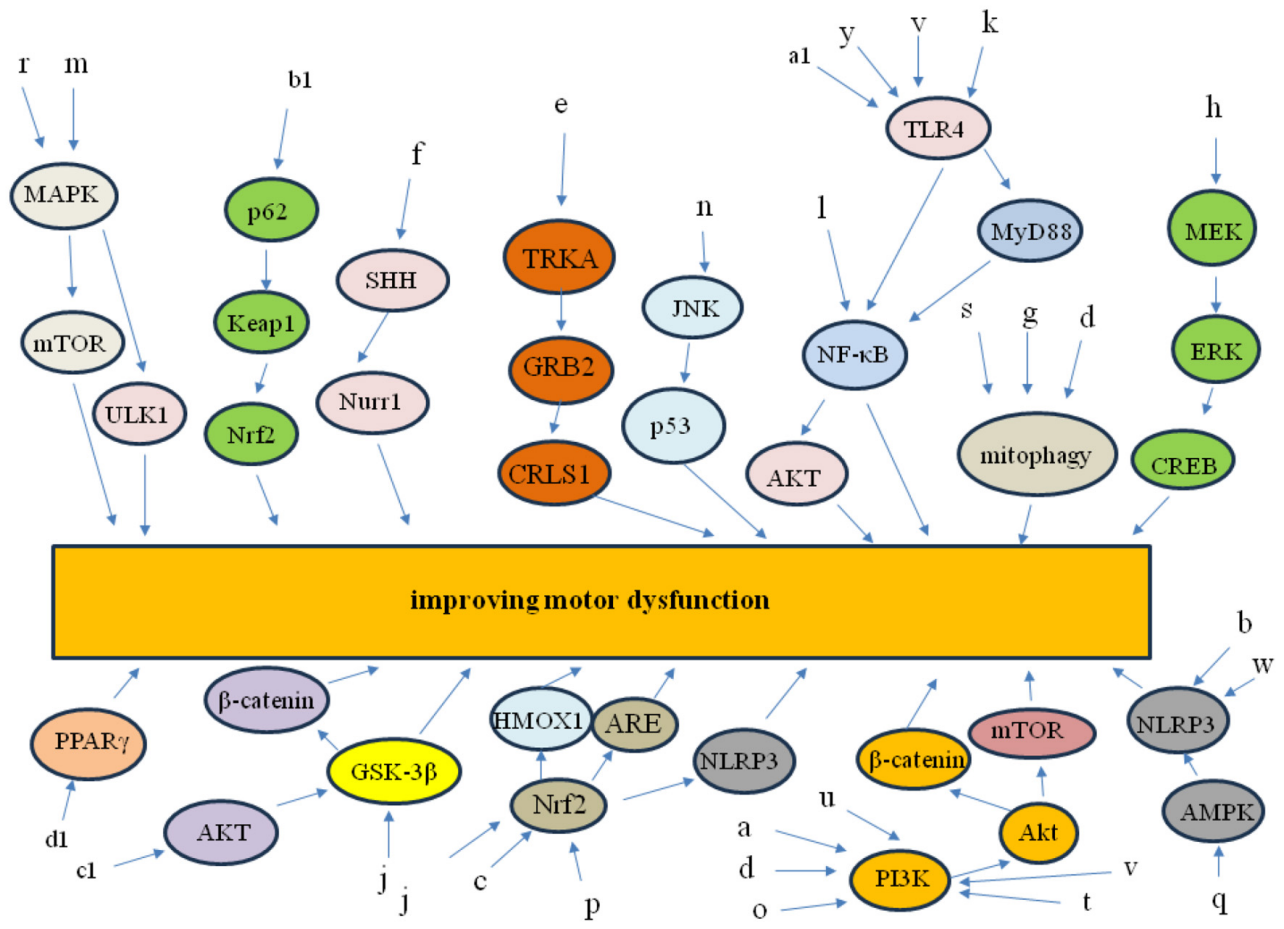
The related mechanisms of the treatment of TCMs in PD.

It has been demonstrated that TCM has a wide range of pharmacological effects, including the regulation of oxidative stress, the reduction of inflammation, and the regulation of immune function. Evidence demonstrated that TCM can protect dopamine neurons, reduce acetylcholinesterase activity in the brain, and improve the activity of energy metabolism in the substantia nigra. These effects can improve the motor symptoms of PD patients and to enhance their quality of life, demonstrating the excellent application potential of TCM in this field. For example, (He Q. K. et al. 2024) investigated the effect of Asiaticoside on the MPTP-induced PD mice. The experimental data demonstrated that Asiaticoside significantly improved the motor dysfunction and increased the number of the loss dopaminergic neurons in PD mice by regulating the expression of NLRP3 protein (He Q. K. et al., 2024). (Pan et al. 2024) demonstrated that Schisandra Decoction exhibited a better neuroprotective effect on MPTP-induced PD mice by increasing the expression of TH protein and promoting the α-syn clearance, and the process was regulated through the PI3K/AKT/mTOR pathway (Pan et al., 2024). And (Sun et al. 2024) demonstrated that Dihuang granule could improve the neurobehavior of PD rats and decrease the damage to dopamine neurons. The mechanisms study indicated that Nrf2/HMOX1 pathway played an important role in this process (Sun et al., 2024). The TCMs have been applied in the treatment of PD in recent years (including modeling methods, therapeutic effect, and mechanism) are summarized in Table 1. There are 56 different types of TCMs have been documented employed in the treatment of DM, including artemisia leaf extract, asiaticoside, and tongtian oral liquid; The animal species utilized in these studies include rats, zebrafish, C. elegans, and mouse. The modeling method employed in these study involved MPTP-induced model, 6-OHDA-induced model, A53T-αSyn induced model, and α-syn PFFs induced model; the therapeutic effect of these TCMs include decreasing α-synuclein, improving motor dysfunction, and improving neuronal synaptic plasticity, and the PI3K/AKT/β-catenin signaling pathway, MEK/ERK/CREB signaling pathway, and Nrf2-NLRP3 signaling pathway are involved in the treatment of TCMs in PD (Table 1, Figure 3).

**Table 1:** The summary of the recent TCM application in PD.

TCM	Animal	Modeling methods	Therapeutic effect	Mechanism	References
Artemisia Leaf extract	Mouse	MPTP-induced	Decreasing α-synuclein	Regulating PI3K/AKT/β-catenin	(Wu et al., 2022)
Asiaticoside	Mouse	MPTP-induced	Improving motor dysfunction	Regulating NLRP3	(He Z. et al., 2024)
morroniside	Mouse	MPTP-induced	Improving motor dysfunction	Regulating Nrf2/ARE	(Li et al., 2023b)
Piperine	Rat	6-OHDA-induced	Reducing α-Syn aggregation	Regulating PI3K/AKT/mTOR	(Yu et al., 2024)
Tongtian oral liquid	Zebrafish	MPTP-induced	Improving motor dysfunction	/	(Dongjie et al., 2022)
Ginsenoside Rg3	Mouse	MPTP-induced	Improving motor performance	Regulating TRKA/GRB2/CRLS1	(Qi et al., 2024a)
Hederagenin	C. elegans	transgenic	Improving motor dysfunction	Regulating mitophagy	(Li X. et al., 2024)
Myricetin	Rat	MPTP-induced	Improving motor impairment	Regulating ferroptosis	(Gu et al., 2024)
Gastrodin	Mouse	MPTP-induced	Improving motor impairment	Regulating MEK/ERK/CREB	(Zhao et al., 2024)
Tianma Gouteng Decoction	Mouse	MPTP-induced	Improving neuronal synaptic plasticity	/	(Lei et al., 2025)
Shisandra Decoction	Mouse	MPTP-induced	Decreasing α-synuclein	Regulating PI3K/AKT/mTOR	(Pan et al., 2024)
Corydaline	Rat	MPTP-induced	Improving the motor coordination	Regulating GSK-3β	(Zhou and Xu, 2024)
Gastrodia elata polysaccharide	Mouse	MPTP-induced	Improving the motor coordination	Regulating TLR4/NF-κB	(Gan et al., 2024)
Bear bile powder	Mouse	MPTP-induced	Improving the motor coordination	Regulating NF-κB/AKT	(Wang L. et al., 2023)
Phillyrin	Mouse	A53T-αSyn-induced	Increasing dopaminergic neurons	Regulating REEP1	(Qi et al., 2024b)
Levistilide A	Mouse	MPTP-induced	Increasing dopaminergic neurons	Regulating AMPK/mTOR	(Zhang M. et al., 2024)
Ganoderic acid A	Mouse	MPTP-induced	Improving motor dysfunction	Regulating NCOA4	(Li Q. et al., 2024)
Huperzine A	Mouse	MPTP-Induced	Improving cognitive abnormalities	/	(Guo et al., 2023)
Buyang Huanwu decoction	Mouse	MPTP-induced	Improving dopaminergic neurons	Regulating liver metabolism	(Hu et al., 2023)
Wolfberry (Lycium barbarum) glycopeptide	Mouse and rat	MPTP and 6-OHDA-induced α-synuclein overexpressed hSNCAA53T mice	Improving motor deficits	/	(Lin X. M. et al., 2025)
Paeoniflorin	Mouse	MPTP-induced	Improving cognitive impairment	Regulating JNK/p53	(He et al., 2022)
Wuzi Yanzong pill	Mouse	MPTP-induced	Improving abnormal motor function	Regulating PI3K/Akt	(Hang et al., 2022)
Baicalin	Mouse	MPTP-induced	Improving motor deficits	Regulating Nrf2/NLRP3	(Huang et al., 2024)
Lignans of Schisandra chinensis (Turcz.) Baill	Mouse	MPTP-induced	Improving pathological damage	Regulating TRPV1/AMPK/NLRP3	(Wang et al., 2024a)
Morin	Mouse	MPTP-induced	Improving behavioral deficit	Regulating AMPK-ULK1	(Wang Z. et al., 2023)
Cholestanol	Mouse	α-syn PFFs-induced	Improving α-syn pathology	Regulating AEP	(Yu et al., 2023)
Uncaria rhynchophylla alkaloid extract	Mouse and rat	MPTP-Induced 6-OHDA-induced	Improving tyrosine hydroxylase expression	Regulating mitophagy	(Hou et al., 2025)
Schisandra chinensis (Turcz.) Baill neutral polysaccharides	Mouse	MPTP-induced	Improving behavioral deficits	Regulating PI3K/AKT/mTOR	(Wang et al., 2024b)
Icaritin	Mouse	MPTP-induced	Improving motor deficits	Regulating ER-PI3K/Akt	(Lin X. et al., 2025)
Berberine	Rat	6-OHDA-induced	Improving depression-like behaviors	/	(Liu et al., 2025)
Cycloastragenol	Mouse	MPTP-induced	Improving motor deficits	Regulating TLR4/NF-κB	(Xiao et al., 2025)
Bilobalide	Mouse	MPTP-induced	Improving astrocytes differentiation	/	(Song et al., 2024)
Bushen Zhichan decoction	Mouse	MPTP-induced	Decreasing death of DA neurons	Regulating EAAT1	(Liu et al., 2024)
Safranal	Mouse	MPTP-induced	Improving motor retardation	Regulating NLRP3	(Yang et al., 2024)
Luteolin	Rat	6-OHDA-induced	Improving motor deficits	Regulating NF-κB/NLRP3	(She et al., 2025)
Z-ligustilide	Mouse	MPTP-induced	Improving motor deficits	Regulating Nrf2/TrxR	(Peng et al., 2024)
ginsenoside CK	Mouse	MPTP-induced	Decreasing apoptosis of dopaminergic neurons	/	(Yang et al., 2025)
glucan from Ganoderma lucidum	Mouse	MPTP-induced	Reducing pathological damage	Regulating TLR4/MyD88/NF-κB	(Chen et al., 2025)
Volatile oils of Schisandra chinensis (Turcz.) Baill	Mouse	MPTP-induced	Decreasing oxidative damage	Regulating Nrf2	(Wang et al., 2025)
Mogroside V and mogrol	Mouse	MPTP-Induced	Enhancing motor coordination	/	(Tang et al., 2024)
Dihuang granule	Mouse	MPTP-Induced 6-OHDA-induced	Decreasing the damage of dopamine neurons	Regulating Nrf2/HMOX1	(Sun et al., 2024)
Astragaloside IV	Rat	6-OHDA-induced	Promoting cell differentiation	Regulating SHH/Nurr1	(Wu et al., 2024)
Canna edulis RS3-resistant starch	Rat	rotenone-induced	Promoting TH-positive neurons	Regulating TLR4/NLRP3	(He Q. K. et al., 2024)
Lilium brownii extracts	Mouse	MPTP-induced	Improving motor dysfunction	Regulating p62/Keap1/Nrf2	(Hui et al., 2024)
Erianin	Mouse	MPTP-induced	Improving motor deficits	Regulating NF-κB/NLRP3	(Yan et al., 2025)
Japanese sake yeast supplement	Zebrafish	rotenone-induced	Decreasing inflammatory responses	/	(Li C. et al., 2024)
Huatan Jieyu Granules	Mouse	MPTP-induced	Decreasing dopaminergic neuronal loss	/	(Zhu et al., 2025)
Perillaldehyde	C. elegans and mouse	MPTP-induced	Improving motor disorders	Regulating G3BP1/2	(Fang et al., 2025)
Gentiopicroside	Mouse	MPTP-induced	Improving motor deficits	Regulating NF-κB/NLRP3/GSDMD	(Shen et al., 2025)
Periplaneta americana L. extract	Mouse	MPTP-induced	Decreasing ROS accumulation	Regulating AKT/GSK3β/β-catenin	(Cao et al., 2024)
Ginkgolide C	Mouse	MPTP-induced	Improving behavioral deficits	Regulating AKT/Nrf2/HO-1	(Gao et al., 2024)
Stem-leaf saponins of Panax notoginseng	Mouse	MPTP-induced	Improving behavioral impairments	Regulating P2Y2R/PI3K/AKT/NF-κB	(Wu et al., 2025)
Duzhong Fang	Mouse	rotenone-induced	Improving cognitive impairment	Regulating sphingolipid metabolism	(Shen et al., 2024)
Xifeng Jiannao pill	Mouse	MPTP-induced	Improving motor function	Regulating MAPK	(Han et al., 2025)
Total Glycosides of Cistanche deserticola	Mouse	MPTP-induced	Improving motor function	/	(Zhai et al., 2024)
Epigallocatechin-3-gallate	Mouse	MPTP-induced	Improving oxidative damage	Regulating PPARγ and Nrf2/HO-1	(Xu et al., 2024)

TCM, traditional Chinese medicine; MPTP, 1-methyl-4-phenyl-1,2,3,6-tetrahydropyridine; 6-OHDA, 6-Hydroxydopamine; TRKA, Tropomyosin-receptor kinase; AMAPK1, mitogen-activated protein kinase 1; mTOR, Mammalian target of rapamycin; CREB, cAMP-response element binding protein; GSK3, glycogen synthase kinase-3; REEP1, receptor accessory protein 1; NCOA4, nuclear receptor coactivator 4; NF-κB, nuclear factor kappa-B; Bcl-2, B-cell lymphoma-2; Nrf2, nuclear factor-erythroid 2 related factor 2; ARE, antioxidant response element; TFAM, mitochondrial transcription factor A; MAPK, mitogen-activated protein kinases; ERK, extracellular regulated protein kinases; JNK, c-Jun N-terminal kinase; ULK1, UNC-51-like kinase 1; AEP, asparginyl endopeptidase; ER, endoplasmic reticulum; EAAT1, polyclonal antibody to excitatory amino acid transporter 1; TrxR, thioredoxin reductase; HIF-1α, hypoxia inducible factor-1α; MyD88, myeloid differentiation primary response protein 88; HMOX1, heme oxygenase 1; SHH, sonic hedgehog; Nurr1, nuclear receptor-related factor 1; KEAP1, Kelch like ECH associated protein 1; G3BP1, Ras-GTPase-activating protein (GAP)-binding protein 1; GSDMD, gasdermin-D; HO-1, heme oxygenase 1; P2Y2R, P2Y2 nucleotide receptor; NLRP3, NOD-like receptor family pyrin domain containing 3; JAK2, Janus kinase; STAT3, signal transduction and transcriptional activator; PI3K, phosphatidylin-ositol-3-kinase; MAPK, mitogen-activated protein kinase; TGFβ1, transforming growth factor-β; FOXO1, forkhead box O1; PPARγ, peroxisome proliferator-activated receptor gamma.

### The clinical application of TCM in PD

As a key step in the development of TCM, clinical trials can confirm the therapeutic effects, reveal adverse reactions and the metabolic processes of TCM, and determine the efficacy and safety of TCM. A total of eight TCMs randomized double-blind placebo-controlled trials have been summarized in the Pubmed database. These include Pingchan granule, Tianqi pingchan granule, Gushen shetuo decoction, Congrong shujing granules, Zishen pingchan granules, Huatan jieyu granules, Bushen yisui, and Ziyin jiangzhuo formula. The treatment period lasts from 12 weeks to 9 months. The following adverse effects that have been identified include including nausea, constipation, dizziness, headache, depression, internal heat, thirsty, and dryness heat, nausea, and diarrhea (Table 2). In details, Gu et al. conducted a study on the efficacy and safety of pingchan granule in PD. The study involved 292 participants. The data demonstrated that pingchan granule significantly improved the MDS-UPDRS-III motor score, freezing of gait, and quality of life (Shen et al., 2024). And Gu et al. also observed that Pingchan granule increased the UPDRS-II, UPDRS-III, and PDSS scores in PD patients, thus demonstrating the efficacy of Pingchan granule in improving depressive symptoms in PD (Xu et al., 2024). Zhang et al. conducted a randomized double-blind placebo-controlled trial to observe the clinical efficacy of tianqi pingchan granule on PD. A total of 100 PD patients were recruited and treated with tianqi pingchan granule for 12 weeks. Data demonstrated that tianqi pingchan granule effectively reduced UDysRS scores (Han et al., 2025). Li et al. conducted a comparative analysis of the efficacy of the gushen shetuo decoction and levodopa and benserazide hydrochloride tablets (control group) on PD patients. The data demonstrated that the gushen shetuo decoction was more effective in reducing the scores on both the Parkinson's Disease Rating Scale (MDS-UPDRS) and the Drooling Severity and Frequency Scale (DSFS) compared with control group. This suggests that the gushen shetuo decoction has a significant impact on the drooling of PD patients (Zhai et al., 2024). Chen et al. administered Congrong shujing granules to the PD patients with Shen essence deficiency. The results demonstrated that Congrong shujing granules decreased the scores of UPDRS sub-II, PDQ-39, and CM syndrome, exhibiting the clinical safety of Congrong shujing granules (Gu et al., 2023). Ning et al. conducted a study on 200 PD patients, dividing them into two groups: one group received Zishen Pingchan granules, while the other received a placebo. Following the treatment of Zishen pingchan granules for 12 weeks, the collected data demonstrated that a decrease in the mean HAMD score and an increase in the PDSS-2 score in patients diagnosed with PD (Zhang M. et al., 2024). Furthermore, (Liu et al. 2020) investigated the efficacy of Huatan Jieyu granules in treatment of PD patients with sleep disorder. The data demonstrated that the total sleep time, non-rapid eye movement 2 (NREM 2) and non-rapid eye movement 3 (NREM 3) all increased significantly (Li et al., 2023a). Jin et al. validated that Bushen Yisui and Ziyin Jiangzhuo could alleviate the constipation symptoms in PD patients following a treatment period of 12 weeks (Gu et al., 2021).

**Table 2:** The recent clinical applications of TCM in PD.

TCM	Clinic trial type	Period	Therapeutic effect	Adverse effect	Country	References
Pingchan granule	Multicenter, randomized, double-blind, placebo-controlled trial	9 months	Improving MDS-UPDRS-III motor score	Nausea; constipation; dizziness; headache	China	(Gu et al., 2023)
Tianqi Pingchan Granule	Randomized double-blind placebo-controlled trial	12 weeks	Reducing UDysRS	Fever	China	(Zhang Y. et al., 2024)
Gushen shetuo decoction	Randomized double-blind placebo-controlled trial	6 months	Reducing motor complications	Not mentioned	China	(Li et al., 2023c)
Pingchan granule	Randomized, double-blind, placebo-controlled trial	24 weeks	Improving UPDRS-II score	Depression	China	(Gu et al., 2021)
Congrong shujing Granules	Randomized, double-blind, placebo-controlled trial	12 weeks	Reducing UPDRS sub-II score, PDQ-39 score	Internal heat, thirsty, and dryness heat	China	(Chen et al., 2020)
Zishen pingchan granules	Multicenter, randomized, double-blind, and placebo-controlled trial	12 weeks	Reducing the depressive symptoms and improving the sleeping quality	Nausea, Diarrhea, Abdominal pain	China	(Ning et al., 2022)
Huatan Jieyu granules	Randomized, double-blind, placebo-controlled trial	6 months	Improving sleep disorder	Mild nausea, bdominal distension	China	(Liu et al., 2020)
Bushen Yisui and Ziyin Jiangzhuo formula	Randomized double-blind placebo-controlled multicenter trial	12 weeks	Improving motor symptoms	Nausea, diarrhea, and headache	China	(Jin et al., 2020)

## Discussion

TCM's unique dialectical treatment and holistic concept, minimal side effects and greater safety make it suitable for long-term use. TCM is mostly derived from natural plants, animals, or minerals, exhibiting the excellent antioxidant stress, anti-inflammatory, and immune regulatory effects, which give it unique advantages in treating many types of chronic diseases including PD. Currently, there has been a surge in the number of preclinical and clinical applications of TCM in PD, exhibit the ideal therapeutic effect in the treatment of PD. However, there are still several issues that require careful consideration. (1) Further research is required on the pharmacological substance basis and the mechanism of TCM. TCM has a long history of application and clear clinical efficacy for many types of diseases. However, due to its complex composition and diverse action stages, it is not yet clear which components (the pharmacological substance basis of TCM) exert the pharmacological effects. Therefore, new technical methods suitable for the research and development of TCM should be developed. And research on internationally recognized standards and specifications for TCM should be conducted urgently. (2) The establishment of quality standards for TCM is paramount. The chemical composition and preparation process of TCM are complex, unstable and lacking in consistency. This has had a detrimental effect on the therapeutic effect of TCM, as well as on its controllability, safety, and effectiveness. Therefore, it is necessary to vigorously conduct research on quality stability and process repeatability. repeatability. Pharmacological markers must be incorporated into the study of TCM quality standards, and fingerprint spectra should be utilized for qualitative control. (3) Multi-center, large-scale standardized clinical trials need to be conducted. The absence of rigorous scientific evaluation of the therapeutic benefits of TCM formulas has impeded their recognition by the global medical community. It is evident that contemporary TCM clinical trials exhibit deficiencies in several key areas, including the absence of randomized controls, limited sample sizes, and the absence of efficacious evaluation methodologies. Therefore, standardized randomized controlled clinical trials that conform to the characteristics of TCM need to be conducted.

In summary, there is considerable potential for the application of TCM in the treatment of PD at both preclinical and clinical levels. the pharmacological substance basis and the mechanism of TCM, the establishment of quality standards for TCM, and the conducting the standardized clinical trials are identified as the main application limitations. The resolution of the aforementioned issues will facilitate significant progress in the application of TCM in PD.
